# The effect of supplementation of multistrain probiotic preparation in combination with vitamins and minerals to the basal diet on the growth performance, carcass traits, and physiological response of broilers

**DOI:** 10.14202/vetworld.2018.240-247

**Published:** 2018-02-24

**Authors:** Sugiharto Sugiharto, Isroli Isroli, Turrini Yudiarti, Endang Widiastuti

**Affiliations:** Department of Animal Science, Faculty of Animal and Agricultural Sciences, Diponegoro University, Semarang, Central Java, Indonesia

**Keywords:** broiler chicks, growth rate, minerals, multistrain probiotics, physiological status, vitamins

## Abstract

**Aim:**

The aim of the present study was to investigate the effect of supplementation of multistrain probiotic preparation in combination with vitamins and minerals to the broiler chicken diets on their growth performance, hematological parameters, and carcass traits.

**Materials and Methods:**

Two hundred and eighty-eight Lohmann 1-day-old broiler chicks were randomly allocated to four groups, i.e., control (without additional supplementation) and three experimental treatments where basal diet was enriched by 0.1%, 0.5%, or 1% of multistrain probiotic preparation in combination with vitamins and minerals, respectively. Blood sampling was conducted on day 28, while the selected organs and eviscerated carcasses were collected on day 42.

**Results:**

Dietary supplementation did not affect (p>0.05) the final body weight, feed intake, and feed conversion ratio of broilers. Supplementation by 0.1% and 0.5% of multistrain probiotic preparation in combination with vitamins and minerals reduced (p≤0.05) heart relative weight of broilers. Dietary supplementation tended (p=0.07) to increase the relative weight of ileum and pancreas of broilers. Supplemented birds had lower (p≤0.05) numbers of leukocytes and eosinophils compared to unsupplemented birds. There were tendencies that supplementation of multistrain probiotics in combination with vitamins and minerals resulted in lower (p=0.07) counts of lymphocytes and heterophils when compared with no supplementation. Supplementation by 0.5% of multistrain probiotics in combination with vitamins and minerals resulted in lower (p≤0.05) serum concentration of uric acid when compared with control. There was no significant effect of dietary supplementation on carcass traits, pH, and drip loss of broiler breast muscles.

**Conclusion:**

Dietary supplementation of commercial broiler feeds with 0.5% of multistrain probiotic preparation in combination with vitamins and minerals was potential to improve digestive functions and physiological status of broiler chickens.

## Introduction

For several decades, Indonesian broiler farmers rely on the commercial feeds produced by feed mill industries. In general, commercial broiler feeds have been formulated to meet the nutritional requirements of the birds. In some cases, the bad feed handling on the farm such as prolonged storage or inadequate storage conditions may, however, impair the nutritional qualities of the feeds. Such condition may cause specific micronutrient deficiencies, especially vitamins and minerals, leading to growth retardation in broilers [1]. To optimize the growth potential of modern broilers in the commercial farms, supplementing the commercial broiler feeds with growth promoters [2], probiotics [3], minerals and vitamins [4-6], green foliage/leaf meals [7], exogenous enzymes [8], or essential amino acids [9] has been conducted. With regard to probiotics, vitamins, and minerals, these components have been known to exert an immune-enhancing effect on poultry [10,11]. Hence, supplementation of commercial broiler feeds with probiotics in combination with vitamins and minerals seemed to be beneficial not only for better growth but also for health of broiler chicks on the commercial farms.

Commercial broiler chickens have been attributed to the excessive fat deposition, especially in the abdomen area [12]. This abdominal fat content may reduce carcass yield of broilers, and any attempt to decrease the abdominal fat pad in broiler chicks will, therefore, be valuable. Feeding probiotics have been reported able to reduce fat content [12] as well as improve carcass traits of broiler chickens [13]. Certain vitamins, for example, tocopherol [14,15] and retinol [16] have also been reported to decrease the abdominal fat content and enhance carcass mass of broilers. Concomitantly, dietary supplementation with minerals such as Mn [17,18], Zn [16], and S [19] was beneficial to decrease the abdominal fat deposition in broiler chickens. Moreover, supplementation of broiler feeds with Cu, Fe, Zn, and Mn improved meat quality of broilers [20]. Considering the above conditions, dietary supplementation of probiotics in combination with selected vitamins and minerals may, therefore, improve the carcass traits of broiler chickens.

It has been shown in our previous study that supplementation of commercial broiler diets with multistrain probiotics in combination with vitamins and minerals increased hemoglobin levels and relative weight of ileum and improved feed efficiency of broiler during the brooding period [21]. Hence, it would be interesting to investigate the effect of such dietary treatment on the performances and physiological responses of broiler chickens at later age. Taken together, the present study aimed to investigate the effect of supplementation of multistrain probiotic preparation in combination with vitamins and minerals to the broiler chicken diets on their growth performance, hematological parameters, and carcass traits.

## Materials and Methods

### Ethical approval

The present study was performed under the standard procedures of rearing and treating of farm animals stated in Law of the Republic of Indonesia number 18, 2009 regarding animal husbandry and health.

### In vivo experiment

A total of 288 unsexed Lohmann (MB–202) 1-day-old broiler chicks (body weight [BW]=45.9±0.50 g; means±standard deviation) were used in the present study. To simulate the commercial condition of broiler chicks handling in Indonesia (exerting growth retardation effect on broiler chicks), all chicks were deprived of feed and water for 24 h after hatching. In an open-sided broiler house, broiler chicks were randomly allocated to one of four treatment groups of 72 chicks each (6 replicates of 12 chicks). The groups were control (without additional supplementation) and three experimental treatments where basal diet was enriched by 0.1%, 0.5%, or 1% of multistrain probiotic preparation in combination with vitamins and minerals, respectively. The feeds were obtained from the local feed mill company. The chemical compositions of feed are presented in Table-1. The feeds and water were provided *ad libitum* throughout the study. The supplement (multistrain probiotic preparation in combination with vitamins and minerals) was added (“on top”) to the basal feeds. The supplement contained 12.10 log cfu/g mixture of *Bacillus* probiotics (i.e., *Bacillus cereus* strain SIIA_Pb_E3, *Bacillus licheniformis* strain FJAT-29133, Bacillus megaterium strain F4-2-27, and Bacillus sp**. 11CM31Y12), 0.100 mg retinol, 0.018 mg cholecalciferol, 0.100 mg alpha-tocopherol, 1200 mg Ca, 750 mg P, 0.08 mg Mg, 0.006 mg Co, 0.045 mg Cu, 0.015 mg Se, 0.180 mg S, 0.010 mg Zn, 0.060 mg KCl, 0.030 mg I, 0.060 mg Fe, and 0.100 mg Mn. The *Bacillus* strains were isolated from the rumen content of cow and showed probiotic potentials [21]. Note that recently there has been a trend to use probiotics isolated from an animal species for another animal species [22,23].

**Table-1:** Chemical compositions of basal diets.^1^

Items (% dry matter)	Composition based on feed label^2^	Composition based on proximate analysis^3^
Moisture	13.0	10.1
Crude protein	21.0–23.0	19.8
Crude fat	5.00	3.39
Crude fiber	5.00	2.09
Crude ash	7.00	3.32

1 Feed was composed of corn, rice bran, fish meal, soybean meal, coconut meal, meat and bone meal, broken wheat, peanut cake, canola, leaf meal, vitamins, calcium, phosphate, and trace minerals. The proportion of each feed ingredient in the ration is company’s confidential information,

2 data were provided by feed mill company,

3 analysis was conducted at the Faculty of Animal and Agricultural Sciences, Diponegoro University. Analysis was conducted in duplicate

At days 4 and 21 of the experiment, the chicks were vaccinated with commercial Newcastle disease virus (NDV) vaccine through eye drops and drinking water, respectively. BW and feed intake of broilers were recorded weekly. At day 28, blood was obtained from the bird’s wing veins and collected in ethylenediaminetetraacetic acid-containing vacutainers for the determination of hematological profile. The rest of the blood was collected in the vacutainers with no anticoagulant. The latter blood was let to clot at room temperature and centrifuged at 2000 rpm for 15 min to obtain serum. The serum was frozen until the determination of antibody titers and biochemical analyses. There was no specific reason for the day of blood sampling in this study (at day 28), and the only rationale was just a technical matter in the field. At day 42, the chicks were slaughtered, de-feathered, and eviscerated. Directly, the selected internal organs were removed, and the relative weight of the empty organs was then determined [24]. In respect to the small intestine, the duodenum was determined as from gizzard outlet to the end of the pancreatic loop, jejunum as from the pancreatic loop to Meckel’s diverticulum, and ileum as from Meckel’s diverticulum to the cecum junction. Samples of breast muscle were also collected for the determination of pH and drip loss.

The hematological profile (complete blood counts) was determined with a hematology analyzer (Prima Fully-auto Hematology Analyzer, PT. Prima Alkesindo Nusantara, Jakarta, Indonesia). Antibody titers to NDV vaccine were determined in serum based on hemagglutination inhibition assay. The titers were expressed as geometric mean titers (log_2_). Total triglyceride in the serum was determined based on an enzymatic colorimetric method using glycerol-3-phosphate oxidase (DiaSys Diagnostic Systems GmbH, Holzheim, Germany). Total cholesterol, high-density lipoprotein, and low-density lipoprotein cholesterol were determined according to an enzymatic colorimetric method with cholesterol oxidase/p-aminophenazone (DiaSys Diagnostic Systems GmbH, Holzheim, Germany). The enzymes of alanine aminotransferase and aspartate aminotransferase were measured spectrophotometrically with a Reflotron system (Roche Diagnostics Corporation, Indianapolis, IN, USA). Serum total protein was determined by photometric test based on the biuret method with the kit (total protein kit, DiaSys Diagnostic Systems GmbH, Holzheim, Germany) according to the manufacturer’s instructions. Albumin in serum was measured by photometric test using bromocresol green (DiaSys Diagnostic Systems GmbH, Holzheim, Germany). Data of globulin were obtained by subtracting albumin values from total protein in serum. Uric acid in the serum was determined according to the enzymatic color test. pH and drip loss of breast meats were measured based on Sugiharto *et al*. [25] with little modifications. The meats were weighed and the pH was determined (using Eutech EcoTestr pH 1, Thermo Fisher, Singapore) around 45 min following slaughter. The breast meats were then put in a Whirl-Pak bag, stored in a refrigerator (5°C), and reweighed after 24 h. The pH of meat was also measured at that time. The meat drip loss was calculated based on the weight loss and presented as a percentage.

### Statistical analysis

Data obtained from the experiment were analyzed based on a completely randomized design by ANOVA using the General Linear Models Procedure in SAS (SAS Institute Inc., Cary, NC, USA). The pen was treated as the experimental unit during the analysis. Significant differences among treatment groups were further analyzed using Duncan’s multiple-range test. A significant level of p≤0.05 was implemented.

## Results

### Performance of broiler chicks

Supplementation of commercial broiler feeds with multistrain probiotic preparation in combination with vitamins and minerals did not affect (p>0.05) the final BW, cumulative FI, and feed conversion ratio (of broiler chicks (Table-2).

**Table-2:** Performances of broiler chickens fed with multistrain probiotic preparation in combination with vitamins and minerals (0-42 days).

Items	Dietary supplementations				SE	p value

	Control	Pro-0.1	Pro-0.5	Pro-1.0				
BW (g)						
Day 0	45.9	45.8	45.9	45.7	0.02	0.90
Day 14	318	324	330	310	4.35	0.25
Day 28	990	1005	1010	974	8.12	0.34
Day 42	1989	2030	2054	2011	43.3	0.75
Cumulative FI (g)						
Day 14	403	395	386	392	6.94	0.30
Day 28	1720	1768	1716	1666	20.8	0.36
Day 42	3725	3922	3893	3689	86.8	0.17
FCR (g/g)						
Day 14	1.47	1.42	1.36	1.48	0.01	0.23
Day 28	1.82	1.84	1.78	1.79	0.01	0.27
Day 42	1.92	1.98	1.94	1.88	0.03	0.22

Control=Birds receiving diet without supplementation, Pro-0.1=Birds receiving diet supplemented with 0.1% of multistrain Probiotic preparation in combination with vitamins and minerals, Pro-0.5=Birds receiving 0.5% of multistrain Probiotic preparation in combination with vitamins and minerals, Pro-1.0=Birds receiving 1% of multistrain Probiotic preparation in combination with vitamins and minerals, SE=Standard error, BW=Body weight, FI=Feed intake, FCR=Feed conversion ratio, n (number of birds per experimental group)=72

### Internal organs of broiler chicks

The data on selected internal organs of broiler chicks are presented in Table-3. Supplementation of commercial diets, especially, with 0.1% and 0.5% of multistrain probiotic preparation in combination with vitamins and minerals reduced (p≤0.05) the relative weight of heart of broiler chicks. Dietary supplementation tended (p=0.07) to increase the relative weight of ileum and pancreas of broilers. No noticeable difference (p>0.05) was observed with regard to other internal organs of broiler chicks.

**Table-3:** Weights of selected internal organs (in relation to live BW) of broiler chickens fed with multistrain Probiotic preparation in combination with vitamins and minerals.

Items (% live BW)	Dietary supplementations				SE	p value

	Control	Pro-0.1	Pro-0.5	Pro-1.0		
Heart	0.45^a^	0.39^b^	0.38^b^	0.44^ab^	0.02	0.05
Liver	2.34	2.33	2.41	2.30	0.11	0.90
Gallbladder	0.04	0.05	0.03	0.04	0.01	0.23
Pro-ventriculus	0.56	0.57	0.54	0.61	0.04	0.60
Gizzard	1.10	1.27	1.09	1.21	0.10	0.50
Spleen	0.11	0.12	0.11	0.13	0.01	0.80
Thymus	0.27	0.29	0.25	0.26	0.03	0.87
Bursa of Fabricius	0.05	0.07	0.05	0.06	0.01	0.22
Duodenum	0.45	0.51	0.49	0.54	0.03	0.18
Jejunum	1.01	1.08	1.03	1.13	0.06	0.54
Ileum	0.81	0.93	1.05	0.95	0.06	0.07
Ceca	0.32	0.34	0.40	0.40	0.03	0.21
Pancreas	0.21	0.26	0.22	0.26	0.01	0.07

Control=Birds receiving diet without supplementation, Pro-0.1=Birds receiving diet supplemented with 0.1% of multistrain Probiotic preparation in combination with vitamins and minerals, Pro-0.5=Birds receiving 0.5% of multistrain Probiotic preparation in combination with vitamins and minerals, Pro-1.0=Birds receiving 1% of multistrain Probiotic preparation in combination with vitamins and minerals, BW=Body weight, SE=Standard error,

a,b Values with different letters within the same row were significantly different, n (number of samples per experimental group)=6

### Hematological parameters of broiler chicks

The data on hematological profile of broilers are presented in Table-4. Broiler chicks offered commercial feeds supplemented with multistrain probiotic preparation in combination with vitamins and minerals had lower (p≤0.05) numbers of leukocytes and eosinophils compared to unsupplemented chicks. There were tendencies that dietary supplementation resulted in lower (p=0.07) counts of lymphocytes and heterophils when compared with no supplementation. The effect of dietary supplementation was not significant with regard to erythrocyte profiles of broilers.

**Table-4:** Hematological traits of broiler chickens fed with multistrain Probiotic preparation in combination with vitamins and minerals.

Items	Dietary supplementations				SE	p value

	Control	Pro-0.1	Pro-0.5	Pro-1.0
Hemoglobin (g/dL)	9.65	9.00	8.37	8.88	0.43	0.16
Erythrocytes (10^6^/µL)	2.06	1.99	1.81	1.94	0.10	0.34
Hematocrit (%)	29.0	28.2	25.9	26.5	1.32	0.35
MCV (fl)	141	142	144	140	2.61	0.76
MCH (pg)	46.8	45.5	46.6	45.8	0.72	0.58
MCHC (g/dL)	33.2	32.0	32.4	32.8	0.63	0.61
Leukocytes (10^3^/µL)	26.3^a^	18.8^b^	20.2^b^	19.6^b^	1.96	0.05
Heterophils (10^3^/µL)	1.12	0.73	0.82	0.67	0.12	0.07
Eosinophils (10^3^/µL)	1.57^a^	1.05^b^	1.18^b^	0.98^b^	0.14	0.03
Lymphocytes (10^3^/µL)	23.6	17.05	18.2	17.9	1.80	0.07
Thrombocytes (10^3^/µL)	17.8	13.7	15.0	15.0	1.99	0.52
H/L ratio	0.05	0.04	0.05	0.04	0.01	0.62

Control=Birds receiving diet without supplementation; Pro-0.1=Birds receiving diet supplemented with 0.1% of multistrain Probiotic preparation in combination with vitamins and minerals, Pro-0.5=Birds receiving 0.5% of multistrain Probiotic preparation in combination with vitamins and minerals; Pro-1.0=Birds receiving 1% of multistrain Probiotic preparation in combination with vitamins and minerals, SE=Standard error, MCV=Mean corpuscular volume, MCH=Mean corpuscular hemoglobin, MCHC=Mean corpuscular hemoglobin concentration, H/L ratio=Heterophils to lymphocytes ratio,

a,b values with different letters within the same row were significantly different, n (number of samples per experimental group)=6

Supplementation with 0.5% of multistrain probiotic preparation in combination with vitamins and minerals in the diets resulted in lower (p≤0.05) concentration of uric acid in the serum of birds when compared with that in unsupplemented birds (Table-5). The other biochemical parameters in the serum of birds were not affected (p>0.05) by the dietary supplementations.

**Table-5:** Serum biochemical traits and antibody titers of broiler chickens fed with multistrain Probiotic preparation in combination with vitamins and minerals.

Items	Dietary supplementations				SE	p value

	Control	Pro-0.1	Pro-0.5	Pro-1.0
AST (U/L)	314	355	332	294	66.2	0.93
ALT (U/L)	7.72	6.82	5.40	5.92	1.17	0.53
Uric acid (g/dL)	5.75^a^	6.52^a^	3.40^b^	4.97^ab^	0.66	0.01
Total triglyceride (g/dL)	82.8	88.5	79.4	83.7	9.39	0.91
Total cholesterol (g/dL)	107	114	115	114	5.37	0.67
LDL (g/dL)	25.4	38.2	41.2	28.1	6.28	0.19
HDL (g/dL)	64.5	53.5	57.8	69.3	6.48	0.35
Total protein (g/dL)	2.67	2.65	2.90	2.77	0.11	0.40
Albumin (g/dL)	1.09	1.09	1.14	1.09	0.04	0.73
Globulin (g/dL)	1.60	1.62	1.76	1.70	0.08	0.47
Antibody titer (Log_2_ GMT)	2.67	3.17	3.67	3.33	0.37	0.32

Control=Birds receiving diet without supplementation, Pro-0.1=Birds receiving diet supplemented with 0.1% of multistrain Probiotic preparation in combination with vitamins and minerals, Pro-0.5=Birds receiving 0.5% of multistrain Probiotic preparation in combination with vitamins and minerals, Pro-1.0=Birds receiving 1% of multistrain Probiotic preparation in combination with vitamins and minerals, SE=Standard error, AST=Aspartate aminotransferase, ALT=Alanine aminotransferase, LDL==Low-density lipoprotein, HDL=High-density lipoprotein, GMT=Geometric mean titer,

a,b values with different letters within the same row were significantly different, n (number of samples per experimental group)=6

### Carcass characteristics of broiler chicks

The data on the effect of dietary supplementation of multistrain probiotics in combination with vitamins and minerals on the carcass characteristics of broilers are presented in Table-6. In general, there was no significant effect of dietary supplementation on the carcass traits, pH, and drip loss of broiler meats.

**Table-6:** Carcass characteristics of broiler chickens fed with multistrain probiotic preparation in combination with vitamins and minerals.

Items	Dietary supplementations				SE	p value

	Control	Pro-0.1	Pro-0.5	Pro-1.0		

% live weight						
Eviscerated carcass	71.7	68.4	68.6	67.6	1.20	0.13
Giblets^1^	3.88	3.99	3.88	3.95	0.17	0.96

**% Eviscerated carcass**						

Breast	37.1	38.3	38.7	37.2	1.01	0.61
Thigh	15.7	15.8	15.9	16.1	0.59	0.98
Drumstick	13.0	13.6	13.1	13.8	0.39	0.52
Wing	10.4	10.7	10.4	10.9	0.27	0.36
Abdominal fat	2.24	1.92	2.36	2.12	0.22	0.49
pH 45 min	5.97	5.87	5.97	5.92	0.10	0.86
pH 24 h	5.72	5.72	5.78	5.73	0.05	0.79
Drip loss (%)	1.38	2.00	1.18	1.58	0.34	0.39

1 Giblets: heart, gizzard, and liver.

Control=Birds receiving diet without supplementation, Pro-0.1=Birds receiving diet supplemented with 0.1% of multistrain probiotic preparation in combination with vitamins and minerals, Pro-0.5=Birds receiving 0.5% of multistrain probiotic preparation in combination with vitamins and minerals; Pro-1.0=Birds receiving 1% of multistrain probiotic preparation in combination with vitamins and minerals, SE=Standard error, pH 45 min=pH of breast meat determined around 45 min following slaughter, pH 24h=pH of breast meat determined around 24 h after slaughter, n (number of samples per experimental group)=6

## Discussion

Extra (“on top”) supplementations of commercial broiler feed with certain components have generally been expected to optimize the genetic potentials of modern broiler strains in terms of growth performance. In the present study, final BW of broilers, however, did not differ among the treatment groups. Our present data were therefore different from those of previously reported [2-6] showing higher final BW in broilers when feeding supplemented commercial feeds. The differences in nature and qualities of supplements and commercial feeds and feed handling on the farm as well as the condition of the trial seemed to be responsible for the above divergent results.

Our present data showed that supplementation with 0.1% and 0.5% of multistrain probiotic preparation in combination with vitamins and minerals decreased the relative weight of heart in broiler chicks. This result was different from Anjum *et al*. [3] reporting no effect of probiotic (Protexin) treatment and Hatab *et al*. [26] showing an increased heart relative weight when feeding probiotics *Bacillus subtilis* and *Enterococcus faecium* to broilers. It has been known that heat stress was associated with the heart enlargement and heart failure in broiler chickens [27,28]. In the present study, the chicks were more likely to experience heat stress as they were raised in an open-sided broiler house with an average temperature of 34°C±1°C (during the day). With regard to the lower relative weight of heart in the supplemented chicks, feeding multistrain probiotics in combination with vitamins and minerals seemed, therefore, to be beneficial for preventing the heart enlargement in broiler chicks raised under high ambient temperature. This inference was concomitant with Khan *et al*. [28] reporting the efficacy of selenium-enriched probiotics in preventing the heart from enlargement (due to enlarged intracellular spaces) in heat-stressed broiler chickens. In the present study, supplementation of commercial feeds with 0.5% of multistrain probiotic preparation in combination with vitamins and minerals resulted in the relatively higher weight of ileum when compared, especially, with control. This finding was consistent with our earlier study in which supplementation with 0.5% of multistrain probiotics in combination with vitamins and minerals increased relative weight of ileum of broiler chicks at the day 12 of age [21]. Indeed, the increased ileum relative weight may be attributed to the improved digestive function of the small intestine of birds [29]. Each component in the supplements (i.e., probiotics, vitamins, and minerals) seemed to contribute for the development of ileum of broilers. Olnood *et al*. [30] have recently reported an increased weight of ileum in broilers at days 21 and 42 of age when feeding probiotic *Lactobacillus*. The mechanism by which probiotics increased ileum relative weight was not exactly known, but probiotic inclusion may increase the thickness of mucus layer and promote the villus development of the intestine [31]. With regard to vitamins and minerals, intestinal morphology and integrity of broilers have been reported to be improved by dietary supplementation of selected vitamins and minerals, for instance, alpha-tocopherol [32] and selenium [33]. These vitamin and mineral may improve the development of the intestinal mucosa and protect enterocytes from proapoptotic oxidant stress [32,33]. There was a tendency in this study that supplementation with multistrain probiotics in combination with vitamins and minerals increased the relative weight of pancreas. This result was in accordance with Olnood *et al*. [34] showing an increased relative weight of the pancreas with feeding probiotic *Lactobacillus johnsonii* to broilers at 21 days of age. Similar to the latter authors, the reason for the increased weight of pancreas was not known in the current study. Taken together, dietary supplementation may improve the development and functionality of ileum and pancreas resulting in a better digestive process and thus growth performance of broiler chickens.

Results in the present study showed that broiler chicks supplemented with multistrain probiotic preparation in combination with vitamins and minerals had lower counts of leukocytes, heterophils, eosinophils, and lymphocytes when compared with the unsupplemented birds. It was apparent in the studies of Shah *et al*. [35] and Akhtar *et al*. [36] that infected broilers had higher numbers of leukocytes and differential leukocytes relative to non-infected birds. This increased leukocytes and differential leukocytes were attributed to the induction of immune responses of the infected broilers against infectious agents [36]. Owing to these earlier data, it may be suggested that supplementation with multistrain probiotics in combination with vitamins and minerals was able to alleviate the potential infections (from the field), resulting in less induction of immune responses of broilers. Our inference was also supported by the fact in the present study that supplemented birds had relatively higher antibody titers against NDV (when compared with the unsupplemented birds) and are thus more protective, especially against NDV. Note that from a clinical point of view, HI titers of <3 log_2_ are generally considered as negative for specific immunity against ND [37], and this condition was observed in the unsupplemented birds in the current trial. A study by Sanda *et al*. [38] revealed that most of feeds available in the market today do not have enough components to stimulate immune responses of broiler chicks. Owing to this fact, supplementation of broiler commercial feeds with multistrain probiotics in combination with vitamins and minerals seems to be beneficial.

In the present study, the concentration of uric acids in serum was lower in birds supplemented with 0.5% of multistrain probiotic preparation in combination with vitamins and minerals when compared, especially, with control. El-Katcha *et al*. [39] reported a negative correlation between the levels of plasma uric acid and protein retention in broiler chickens. Given that we did not measure nitrogen retention in this study, it was therefore difficult to infer that the lower level of serum uric acid in supplemented birds was associated with the higher retention of protein in the body of birds, as the final BW and serum total protein levels were not substantially different among the treatment groups. Besides the indicator of protein metabolism, serum uric acid concentration has been used to indicate infections and toxicosis in farm animals. Lin *et al*. [40] reported that serum uric acid concentration was found higher in infected (with nephropathogenic infectious bronchitis virus) growing layers as compared to non-infected ones. Concomitant with this, serum concentration of uric acid was higher in broiler chickens fed mycotoxins [41]. Ridi and Tallima [42] in their review pointed out that uric acid is the major antioxidant molecule in the blood, which is essential to counteract oxidative damage due to infections and toxicosis. Uric acid has also been reported able to stimulate the immune responses of animals against the invasion of pathogenic microorganisms [43]. Overall, supplementation of commercial broiler feeds with multistrain probiotics seemed beneficial to reduce the invasion of infectious agents [44], resulting in less production of uric acids (as part of defense mechanisms against infections). Concomitant with our result, Kalorey *et al*. [41] reported that polyherbal preparation could inhibit the rise in serum uric acid concentration in aflatoxin fed broiler chicks.

In the current study, extra (“on top”) supplementation of commercial diets with multistrain probiotic preparation in combination with vitamins and minerals had no significant effect on fat deposition and carcass characteristics of broiler chickens. With regard to probiotics, the previous studies revealed the role of probiotics in reducing the fatness [12] and improving the carcass traits [13] of modern broiler strains. Conversely, some studies failed to show such effect, and for instance, Toghyani *et al*. [45] reported no effect of probiotic (Protoxin™) on abdominal fat content and carcass yield of broilers. Similarly, Haščík *et al*. [46] did not find any effect of probiotics (*Lactobacillus fermentum*) on abdominal fat and carcass characteristics of broiler chicks. The different types of probiotic microorganisms, strains of broiler chicks, and conditions of trials seemed to be responsible for the above divergent results. Similar to probiotics, the role of vitamins and minerals in affecting fat deposition and carcass characteristics of broiler chickens was minimum in the current study. Our result was therefore different from the data mentioned in the previous section. The different kinds and levels of vitamins and minerals used in the experiment as well as the conditions of the trials seemed to be responsible for the divergent results.

## Conclusion

Dietary supplementation of commercial broiler feeds with 0.5% of multistrain probiotic preparation in combination with vitamins and minerals was potential to improve digestive functions and physiological status of broiler chickens.

## Authors’ Contributions

SS designed, performed the work, and wrote the manuscript, TY and EW performed the work and revised the manuscript, and II performed the data analysis. All authors read and approved the final manuscript.
